# Proceedings of the Cincinnati Medical Society

**Published:** 1880-02

**Authors:** 


### PROCEEDINGS OF THE CINCINNATI MEDICAL SOCIETY.

Cincinnati, Jan. 13, 1880.

Dr. William B. Davis read a paper on intestinal obstruction.

Dr. Bruhl asked if any of the members had had experience with crude mercury.

Dr. Carson, when a student, saw his preceptor administer quicksilver without any beneficial results. The patient died. He had treated two cases of occlusion, with fæcal vomiting, with opium in large doses, and both recovered. He thought well of warm water enemata in early stages, but would give in addition full doses of opium.

Dr. Stanton treated one case successfully during the late war with opium alone. He thought that great harm was often done in these cases by purgatives.

Dr. Wenning—Case VI. of Dr. Davis was undoubtedly saved by the warm water enemata. He considered it necessary for the physician to use the syringe himself, and throw the water high up by the use of a large-sized gum catheter.

Dr. C. P. Judkins treated a lady for fæcal impaction with large enemata of warm water. Impaction gave way, and the patient discharged immense quantities of strawberry seeds, and made a good recovery. He had thought that strawberries were laxative in their influence.

Dr. Davis—Case V. was one of impaction from strawberry seeds. Dr. Carver, the attending physician, whose practice extends over a section of country where strawberries are extensively cultivated for the market, informed him that he had treated six cases of impaction from strawberry seeds.

Dr. Comegys said that he had met with four ceses of intestinal obstruction during the past four years, in each of which the patients were apparently relieved by very large injections of tepid water, repeated as often as needed. At the same time opium and morphia were used, the latter hypodermically. Three of the cases were consultations; the fourth was his own patient, with regard to whom he made the following brief note: Morris B., aged thirty-three, an attorney, general health good, was taken with general bad feeling, accompanied with slight fever, on Friday evening, May 19, 1877. He thought it all due to improper food. I saw him next morning, 20th, and found him with some tenderness in right iliac fossa, and a temperature of 101.5° F. I ordered a mild purgative. At my evening visit found that the bowels had not been moved and the local pain was increased. I suspected typhlitis, and prescribed six leeches for the region of pain, and small, repeated doses of calomel and jalap. May 21st, on my call, found that the medicine had not acted and the pain was not alleviated. Ordered castor oil and turpentine diffused in a pint of water as an enema. No action of the bowels following, I

concluded that I had to deal with an obstruction of the intestinal tract. I now gave one-third of a grain of morphia hypodermically, and injected the bowel with as much tepid water as he could bear—between two and three quarts. This was repeated twice, also the morphia, during the night. All distress of the patient had subsided under the use of the morphia. On the next morning (22d) I began the use of castor oil, tablespoonful every forty-five minutes for three doses. There were some fæcal discharges during the day, accompanied by gas. On the 23d I gave him another large injection, which was followed in due time by an abundant fæcal evacuation. It was quite a week before he could resume his office duties. I think that the physician should always administer the injections. I have great confidence in the use of huge injections of tepid water. Of course there are obstructions in which they could bring no relief.

Dr. Carson said: The subj·ct of intestinal obstruction is always practical and interesting. Notwithstanding the variety of causes, the modern treatment of it is comparatively simple. In some previous remarks he had illustrated the treatment of cases by the use of opium or hypodermic injections. In the present discussion very little had been said about the surgical treatment, and yet it has been of late attracting a good deal of attention, as reference to hospital reports, society proceedings, etc., will show. I shall refer to two cases, the review of one of which and the history of the other as reported will serve to introduce the question of surgical interference. My own experience is too small to be of any interest, as I never saw the operation but once, and it was fatal. The case to which I referred as coming within my observation was a woman about forty years of age, whose general history of good health had never been disturbed much except by a number of confinements. Near five years ago there began the symptoms which, at several different periods up to the time of her death, had recurred with the same general features of fæcal impaction and pain, with relief by opiates and laxatives. There was evidence, satisfactory because she was under intelligent observation all the time, that the tumor-like mass had never entirely disappeared. The fear of malignant growth had been present in the minds of her physicians after her later attacks, as the mass was large, irregular and quite indurated, and yet the duration of the case argued strongly against the view. The last attack, of which she died, was one of severe pain loss of tumor enlargement, nausea, fæcal vomiting and distension, with no satisfactory action of the bowels after several cautious attempts with laxatives. The treatment was principally the opium and enemata plan. A post-mortem showed no peritonitis, great distention of the small bowels above a point about five feet above the ileo-cœcal valve. At that point there was felt an obstructing mass lying on the left side of the abdomen, whereas all of the previous impactions were located in the region and direction of the ascending colon. On section through the bowel at the point of the obstructing mass (which allowed nothing to pass) there was removed a body about one and three quarters by two and one half inches in measurement, with the color and general external appearance of a very large gall stone. What the more minute examination of its structure has since shown I do not know, as I have not seen her physician since. There were evidences that the concretion had passed by ulcerative action from the gall-bladder directly into the duodenum, as there were adhesions between the bowel and gall-bladder, and also a cicatrix on the mucous membrane of the duodenum, though nothing in her history could have suggested the probability of such a thing.

In the last (the twelfth) volume of the Clinical Society's Transactions is related a case parallel in several repects with this one, especially as to the cause of the obstruction and the probable mode of entrance of the concretion into the bowel. Mr. Bryant concluded to operate; he found the concretion, a gall-stone that weighed two hundred and thirty-eight grains, and measured one and seven-eighths by one and one-eighth inches, and three and one-quarter inches in circumference. His case was more unpromising than the one we relate, because of peritonitis. There was reason, however, to attribute our case to some cause several years in action, and it did not therefore come so clearly within Mr. Bryant's emphatic dictum, which he lays down in the following language :

" I hold that in all cases of acute intestinal obstruction that resist medical treatment, operative relief should be resorted to as soon as a reasonable diagnosis has been made, and that, as in a desperate case of strangulated external hernia, an exploratory operation should be performed in most cases. I believe that the physician or surgeon has no right to waste time whilst he is speculating as to the exact seat of the obstruction or precise cause by which it has been brought about, since there is no room to doubt that during this ruminating process the prospects of recovery are rapidly diminishing. Indeed, I believe a surgeon may as well delay in cutting the rope in a case of suicide by hanging whilst he is speculating as to the influences which have led the man to perpetrate the act, as to delay operative interference in a case of acute intestinal obstruction, with the hope that he will be able to make a scientific diagnosis of the case, or that something will turn up by which relief may be obtained." " I maintain that it is not required of the surgeon to diagnosticate the precise cause of the obstruction, so long as the diagnosis of its existence can be determined; and do not think, because such cases as these occasionally recover without operative treatment, we should forget that a large majority die miserably, unrelieved. I plead, therefore, for the majority."

Many of us have felt just such helplessness as Mr. Bryant here records in the presence of such cases, and I have felt that perhaps we have not given as much weight to such language as Mr. Bryant uses as the facts required. The application of his rule to our case would have given the patient a better chance than persistence in the course which we pursued. I repeat that the surgical treatment of obstruction seems to be more prominent, and deserves consideration in this discussion.

Dr. Davis—The difficulty of determining the cause and locality of the occlusion is a great stumbling-block in the way of surgical interference. An old writer has said that in intestinal obstruction we cannot accept any diagnosis not certified by a post-mortem examination. Brinton does not think gastrotomy justifiable, while equally weighty authorities advise it under certain restrictions, believing that it gives the patient one more chance. The introduction of a fine trocar into the distended intestine gives great relief by allowing a discharge of the gas. This operation may be done repeatedly if the trocar is properly disinfected. Larger trocars have been thrust into the non-resonant parts of the abdomen for the removal of the liquid fæces above the obstruction.

Dr. N. P. Dandridge—In the discussion of a subject like the obstruction of the bowels, there is always difficulty in making comprehensive and general statements, from the fact that the conditions under which obstruction exists are so entirely different in different cases. This difficulty is especially apparent in the discussion of any plan of treatment which shall be generally applicable where obstruction is the prominent symptom to be overcome. The variety of conditions under which these cases occur is well illustrated by Dr. Davis' paper. Examples of peritonitis, invagination, twist and impaction were all presented in the six cases reported. The diagnosis in individual cases of the exact seat and cause of the trouble is notoriously a most difficult and often an impossible task. It is this difficulty in diagnosis of the essential condition present that makes it impossible to advocate with confidence any routine course as suitable to all cases. Each individual case must be considered for itself, and a correct diagnosis must precede a judicious treatment. Two general rules, however, seem to be applicable to all of these cases—first, the full and free use of opium to allay pain, and to secure complete rest to the bowels, and, secondly, the contra-indication to the use of active purgatives by the mouth. These two factors in the treatment naturally supplement one another. As for the use of injections into the bowels, their effect must depend upon the situation and character of the obstruction. In general terms it may be stated that where the obstruction is above the ileo-cæcal valve injections are not likely to be directly efficient; not that the emptying the lower bowel may not prove beneficial, but in such conditions as impacted gall-stones, twists, invagination, or incarceration from old adhesions, relief or any effect whatever from injections must be regarded as fortuitous, and not to be relied upon. Even where the obstruction exists low down in the large intestines, injections may only aggravate the trouble. Of this I saw a notable example in the mortuary of the Cincinnati Hospital some time ago, in the case of a woman suffering from obstruction, where large injections of water had been thrown up the bowel and been retained. At the autopsy, a twist in the sigmoid flexure was found to exist, giving rise to a valve-like arrangement which permitted the injected fluid to pass up the bowel, but prevented its return. The sigmoid flexure was enormously distended with water, and filled a large part of the abdominal cavity.

In this connection I may mention two autopsies which I have recently seen. In one, a case of acute onset, which ran its course in a week, a section of the upper part of the ileum twenty inches long was found, of a dark mahogany color, somewhat softened, and its vitality completely compromised. There was entire absence of peritonitis, no twists or bands, and absolutely nothing which would explain the manner in which the circulation had been cut off. The only explanation given was the probable existence of a twist completely compressing the vessels of the involved mesentery, which had become untwisted, possibly by some accidental movement, before death. In another case a loop of intestine had slipped under an old adhesion and become incarcerated. In neither of these cases would an injection have proved of the slightest value in overcoming the especial lesion present.

Dr. Davis—Dr. Dandridge takes the position that enemata can be of no service except for emptying the lower bowel, when the occlusion is above the ileo-cæcal valve. Leichtenstern distinctly states that the ileo-cæcal valve can be so relaxed by large doses of opium or chloroform as to open the way for injected water to reach the point of occlusion, and he particularly mentions twist, knot, and invagination as conditions of obstruction where enemata will be most serviceable. Brinton, either directly or indirectly, expresses the same views.

Dr. Goode gave the particulars of a case to which he had been called in consultation, in which the obstruction had existed for three days. There were constipation, fæcal vomiting, and intense pain below the umbilicus. Opium treatment was résorted to, morphia being given hypodermically in doses of two-thirds of a grain as often as the necessities of the case demanded. No point of induration or tumor could be discovered, on account of the extreme pain, tympanites, and the fact that the inflamed portion of the intestine was partly in the pelvic cavity.

On post-mortem examination he found invagination of four or five inches of the ileum. The peritoneum was much inflamed, and the invaginated portion of the intestine sphacelated. In this case surgical procedure might have relieved the invagination if resorted to early.—*Boston Med. and Surg. Journal.*

